# Henoch-Schönlein purpura with intracerebral haemorrhage in an adult patient: a case report

**DOI:** 10.1186/1752-1947-2-200

**Published:** 2008-06-12

**Authors:** Lazarus Karamadoukis, Linmarie Ludeman, Anthony J Williams

**Affiliations:** 1 The Richard Bright Renal Unit, Southmead Hospital, Westbury upon Trym, Bristol, BS10 5NB, UK; 2 Department of Histopathology, Gloucestershire Royal Hospital, Great Western Road, Gloucester, GL1 3NN, UK; 3 Cotswold Dialysis Centre, Gloucestershire Royal Hospital, Great Western Road, Gloucester, GL1 3NN, UK

## Abstract

**Introduction:**

Henoch-Schönlein purpura is a small vessel vasculitis that affects mainly the skin, joints, gastrointestinal tract and kidneys. The central nervous system is also occasionally affected, although the majority of patients experience only mild symptoms such as headaches and behavioural changes. Intracerebral haemorrhage is a rare complication of Henoch-Schönlein purpura that so far has mainly been described in children and young adolescence.

**Case presentation:**

We describe a 42-year-old man with Henoch-Schönlein purpura who developed an acute intracerebral haemorrhage that coincided with a reactivation of his vasculitis and the development of renal failure following discontinuation of steroids. In this patient, both the Henoch-Schönlein purpura and his neurological symptoms were successfully treated with intravenous cyclophosphamide and methylprednisolone, followed by a short course of oral cyclophosphamide and long-term oral prednisolone. His renal function also recovered sufficiently not to require renal replacement therapy.

**Conclusion:**

The management of Henoch-Schönlein nephritis remains unclear, especially in the presence of severe complications such as intracerebral haemorrhage. We describe a successful outcome in such a patient.

## Introduction

Henoch-Schönlein purpura (HSP) is a small vessel vasculitis characterized by IgA1 deposition in the renal mesangium and in the blood vessels. It is seen most frequently in early childhood, although it can occur at any age [1,2]. It is usually preceded by upper respiratory tract infections, having a peak incidence in the autumn and winter [1,2]. In most cases it is a self-limiting disorder and tends to resolve within 1 month of presentation, although it can re-occur in a third of cases [1,2].

HSP affects mainly the skin, joints, gastrointestinal tract and kidneys [1-3]. The severity of symptoms is usually worse in older patients, who tend to have more frequent skin, joint and renal involvement [2]. The central nervous system is also occasionally affected, although the majority of patients experience only mild symptoms such as headaches and behavioural changes [3]. More serious neurological complications are rare and include seizures, cranial or peripheral neuropathies, intracerebral haemorrhage and encephalopathy [1,3]. We describe a man with HSP who developed an acute intracerebral haemorrhage that coincided with a reactivation of his vasculitis.

## Case presentation

**Figure 1:**
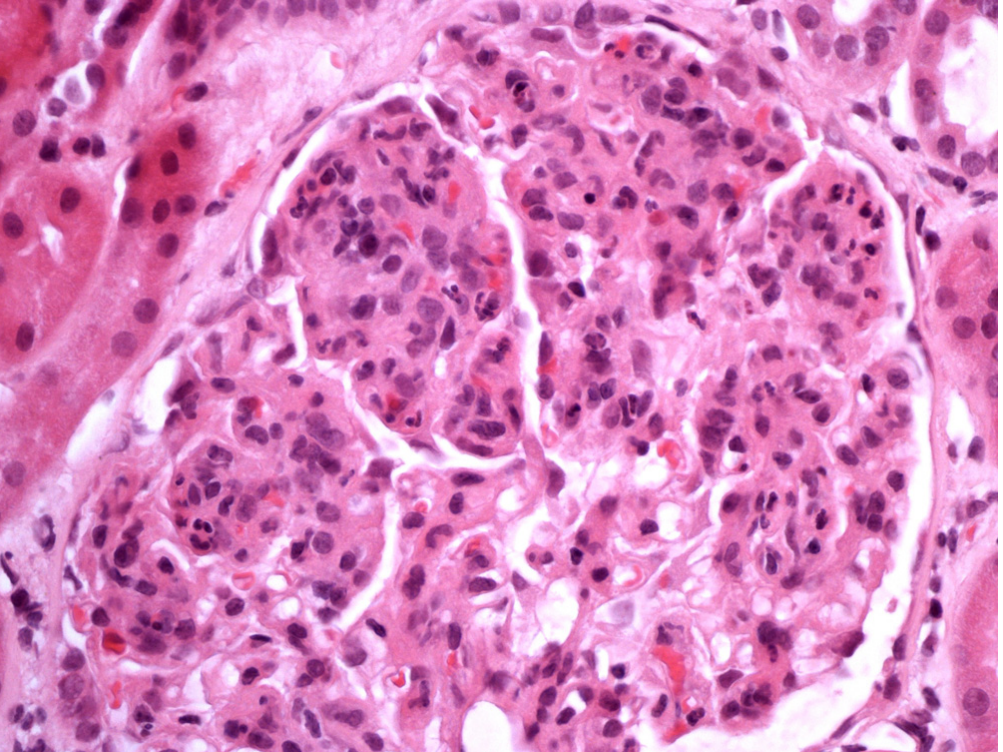
Glomerulus demonstrating increased mesangial cellularity and endocapillary proliferation.

**Figure 2:**
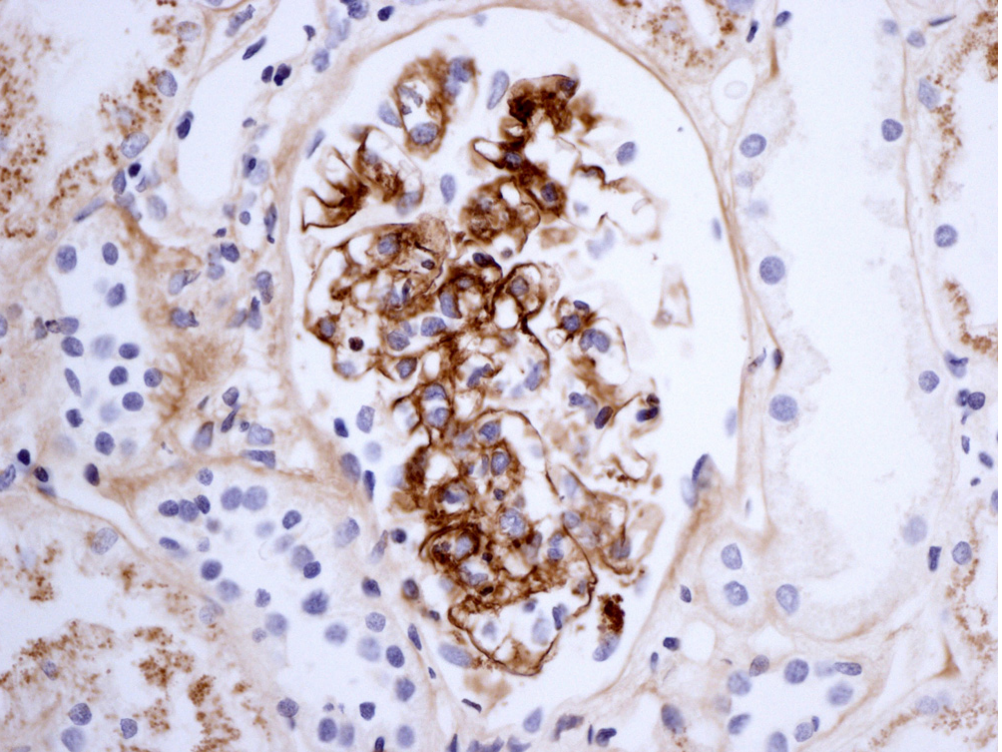
Immunostaining for IgA.

A 42-year-old man presented with acute onset of a vasculitic rash on his buttocks and feet, abdominal pain and arthralgia. This had been preceded by an episode of sore throat 10 days previously. He was found to be hypertensive with a blood pressure (BP) of 158/104 mmHg and he had marked peripheral oedema. He was not known to be hypertensive and he had no other past medical history. Urine dipstick was positive for 3+ of blood and 4+ of protein. His urinary protein to creatinine ratio (PCR) was elevated at 283. There was a significant increase of his serum creatinine from 108 to 152 μmol/l. He received three doses of intravenous methylprednisolone 500 mg, followed by oral cyclophosphamide 100 mg once daily and prednisolone 60 mg once daily. Antineutrophil cytoplasmic antibodies and antinuclear antibodies were negative. A renal biopsy was performed which showed diffuse proliferative glomerulonephritis with marked IgA staining compatible with Henoch-Schönlein nephritis (Figures 1 and 2). Over the following few days his serum creatinine increased to 300 μmol/l, but subsequently returned to 151 μmol/l. His rash and the other systemic features also resolved. In the absence of any crescents in the renal biopsy, the cyclophosphamide was discontinued and he was discharged home on oral prednisolone 50 mg daily. However, because of severe indigestion despite taking omeprazole 20 mg once daily, the prednisolone was reduced to 30 mg once daily soon after.

Upon review 2 weeks later the patient's rash had returned and he remained hypertensive with a BP of 145/95 mmHg. He was therefore prescribed azathioprine 100 mg and ramipril 1.25 mg daily and the prednisolone was increased again to 40 mg once daily. Unfortunately he was again unable to tolerate the increased dose of steroids due to gastrointestinal side effects and the prednisolone was therefore discontinued.

**Figure 3:**
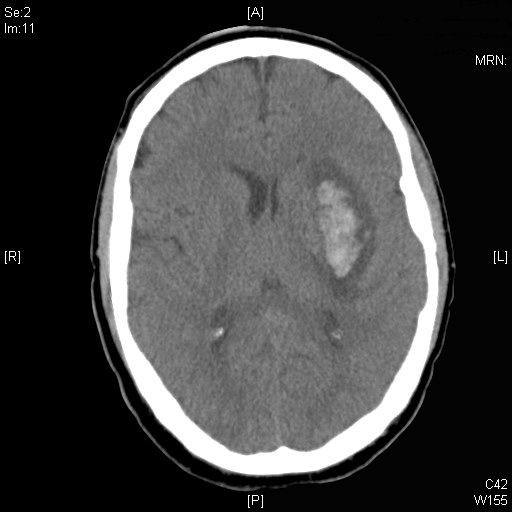
Computed tomography scan of the head demonstrating left internal capsule haemorrhage.

One week later he presented to the accident and emergency department with sudden onset of a severe headache, right-sided weakness and expressive dysphasia, followed by a generalized seizure. He was hypertensive with a BP of 194/115 but with no papilloedema. Initial investigations showed a serum creatinine concentration of 438 μmol/l, haemoglobin of 11.2 g/dl, a white cell count of 10.9 × 10^9^/litre, a platelet count of 2269 × 10^9^/litre, prothrombin time of 14 seconds and activated partial thromboplastin time of 28 seconds. He was transferred to the intensive therapy unit where he was intubated and ventilated for 24 hours. A computed tomography scan of his head confirmed a large left internal capsule haemorrhage, but showed no mass effect (Figure 3). He was treated with four doses of intravenous methylprednisolone 500 mg and one dose of intravenous cyclophosphamide 750 mg, which was followed by oral prednisolone 40 mg once daily and cyclophosphamide 100 mg once daily. Although his dysphasia and weakness were improving, his renal function declined rapidly and he required haemodialysis.

The cyclophosphamide was discontinued 2 weeks later and the patient was discharged home on oral prednisolone 25 mg once daily. Four weeks after the acute admission he had made an almost complete recovery from the intracerebral haemorrhage apart from some mild dysphasia and his renal function improved enough not to need renal replacement therapy. One year later he remains well and dialysis-independent with a serum creatinine of 295 μmol/l, but has continued taking oral prednisolone 10 mg once daily, a dose which is gradually being reduced. His PCR has improved to 87 and urine dipstick has remained positive for blood 1+ and protein 1+.

## Discussion

Intracerebral haemorrhage is a rare complication of HSP that so far has mainly been described in children and young adolescents [3-9]. Apart from this case, there is only one other published report in an older patient, to the best of our knowledge [10]. Such patients may develop acute elevations in BP, and this is considered to be the primary mechanism of intracerebral haemorrhage [11]. However, other possible causes include the presence of cerebral vasculitis and the increased risk of haemorrhagic complications seen in patients with HSP. The increased risk of bleeding in HSP has been attributed to reduced levels of factor XIII [4] and prothrombin [5]. Other reported sites of bleeding in patients with HSP include the gastrointestinal tract, lungs, testicles and bladder [6]. Intracerebral haemorrhage in patients with HSP has been successfully treated in the past with surgical evacuation of the haematoma [7], steroids [8] or plasmapheresis if cerebral vasculitis is confirmed by magnetic resonance imaging [9]. The underlying coagulopathy should also be corrected [4,5].

HSP nephritis may affect as many as 80% of adult patients with HSP and approximately 30% of them will develop chronic kidney disease [2]. Adverse prognostic indicators for progression of HSP nephritis are the presence of crescents on biopsy, more than 1 g of proteinuria per 24 hours and renal impairment on presentation [2]. The optimal treatment of HSP nephritis remains unclear, because of the lack of prospective randomized trials. Intravenous pulse methylprednisolone followed by oral steroids has been shown to be effective in the management of severe HSP nephritis [12]. Other possible treatment regimens for severe HSP nephritis include a combination of corticosteroids with cyclophosphamide, azathioprine or cyclosporin [1,2].

## Conclusion

Intracerebral haemorrhage is a rare complication of HSP that may be caused by acute hypertension, cerebral vasculitis, and the increased risk of bleeding observed in this disorder. Although our patient was severely hypertensive at the time of presentation, both the HSP nephritis and his neurological symptoms were successfully treated with intravenous cyclophosphamide and methylprednisolone, followed by a short course of oral cyclophosphamide and long-term oral prednisolone. His renal function recovered enough not to require renal replacement therapy.

## Abbreviations

BP: blood pressure; HSP: Henoch-Schönlein purpura; PCR: protein to creatinine ratio.

## Competing interests

The authors declare that they have no competing interests.

## Authors' contributions

LK wrote the initial draft of the manuscript. LL provided the renal biopsy pictures and wrote the legends. AJW revised and help to write the manuscript. All authors read and approved the final manuscript.

## Consent

Written informed consent was obtained from the patient for publication of this case report and any accompanying images. A copy of the written consent is available for review by the Editor-in-Chief of this journal.
